# Advancements in *Litchi chinensis* Peel Processing: A Scientific Review of Drying, Extraction, and Isolation of Its Bioactive Compounds

**DOI:** 10.3390/foods13101461

**Published:** 2024-05-09

**Authors:** Christian Iván Cano-Gómez, Angel Josabad Alonso-Castro, Candy Carranza-Alvarez, Jorge E. Wong-Paz

**Affiliations:** 1 Facultad de Estudios Profesionales Zona Huasteca, Universidad Autónoma de San Luis Potosí, Cd. Valles, San Luis Potosi 79080, Mexico; a363206@alumnos.uaslp.mx (C.I.C.-G.); candy.carranza@uaslp.mx (C.C.-A.); 2 Departamento de Farmacia, Universidad de Guanajuato, Noria Alta, Colonia Noria Alta Guanajuato, Guanajuato 36250, Mexico; angeljosabad@ugto.mx

**Keywords:** *Litchi chinensis*, bioactive compounds, drying methods, biological properties

## Abstract

This article systematically reviews the advancements in processing litchi peel (*Litchi chinensis*), emphasizing drying, extraction, purification methods, and the potential of bioactive compounds obtained from litchi peel. This work also highlights the impact of various drying techniques on phytochemical profiles, focusing on how methods such as hot air and freeze-drying affect the preservation of bioactive compounds. The study delves into extraction methods, detailing how different solvents and techniques influence the efficiency of extracting bioactive compounds from litchi peel. Furthermore, the purification and characterization of active compounds, showcasing the role of chromatographic techniques in isolating specific bioactive molecules, is discussed. Biological properties and mechanisms of action, such as antioxidant, antihyperglycemic, cardioprotective, hepatoprotective, anti-atherosclerotic, and anticancer activities, are reviewed, providing insight into the potential health benefits of litchi peel compounds. This review highlights the importance of optimizing and selecting accurate drying and extraction methods to maximize the therapeutic effects of litchi peel and its bioactive compounds. This review also reveals the broad pharmacological potential of the isolated compounds, underscoring the need for further research to discover their specific actions and health benefits.

## 1. Introduction

Litchi (*Litchi chinensis*), a tropical fruit native to China, is cultivated worldwide in warm climates and is an important fruit crop with an export potential playing a significant role in its economy [1,2]. The residues generated from litchi harvest or processing represent a source of phytochemical compounds with biological activities [3]. Litchi peel is used for the empirical treatment of diarrhea, cough, fever, hypertension, and other diseases [4]. Originating as a by-product of the litchi fruit, often discarded as waste, the peel has emerged as a significant source of active compounds. Extensive research reveals its rich phytochemical diversity, which includes simple flavonols, sesquiterpenes, phenolic acids, and proanthocyanidins, contributing to its wide range of medicinal properties such as antioxidative, antidiabetic, cancer preventive, and anti-inflammatory effects [5]. Finding bioactive compounds derived from plant sources, particularly those found in the litchi peel, represents a frontier in the quest for natural antioxidants, antihyperglycemic agents, cardioprotective compounds, and other medicinal uses. This journey begins with the extraction of a complex mixture of phytochemicals, which necessitate the isolation and purification of compounds with pharmacological interest [6,7]. Litchi peel, often discarded as waste, emerges as a significant source of active compounds, with studies revealing its rich phytochemical diversity [8,9]. A crucial initial step in harnessing these compounds is the drying process, which significantly influences the preservation and concentration of bioactive compounds. Techniques such as hot air and freeze-drying are carefully selected to maintain the integrity of these compounds, affecting their subsequent extraction and the overall effectiveness of the derived products [10]. The drying of litchi peel is not just a precursor but a determinant in the quality of extracts obtained, paving the way for effective compound isolation. Following drying, the extraction process plays a pivotal role. Methods ranging from conventional solvent extraction to advanced techniques like supercritical fluid extraction and ultrasound-assisted extraction are employed, each with its own set of parameters designed to optimize the yield of bioactive compounds. This stage is crucial, as the choice of extraction method and solvent can significantly impact the phytochemical profile of the extract, thereby affecting its biological efficacy [11]. The isolated compounds undergo purification, based on chromatographic techniques such as size exclusion, silica gel, and affinity chromatography. These purification processes are crucial for isolating specific bioactive molecules, allowing for a detailed investigation of their properties and potential health benefits. The characterization of bioactive compounds, including flavonoids, proanthocyanidins, and anthocyanins, marks a significant advancement in the field, highlighting the indispensable role of chromatography in phytochemical research [12,13]. The bioactivities of these extracts, such as antioxidant, antihyperglycemic, cardioprotective, hepatoprotective, antiatherosclerotic, and anticancer activities, have been meticulously reviewed through *in vitro* and *in vivo* assays. These studies showed the potential therapeutic applications of litchi peel compounds and addressed their mechanisms of action, providing a foundation for future research and development [11]. The comprehensive study of bioactive compounds in litchi peel, from drying and extraction to purification and characterization, reveals a complex yet promising field of research. The therapeutic potential of these compounds underscores the importance of continuing to refine processing techniques and delve deeper into their mechanisms of action.

Further research into litchi peel holds the promise of unlocking new health benefits, releasing future investigations, potentially leading to novel therapeutic agents and a deeper understanding of plant-based bioactive compounds. Exploring this underutilized resource could pave the way for significant advancements in dietary supplements, pharmaceuticals, and nutraceuticals, highlighting the need for ongoing research and development in this area.

## 2. Methods of Drying Litchi Peel

**Figure 1:**
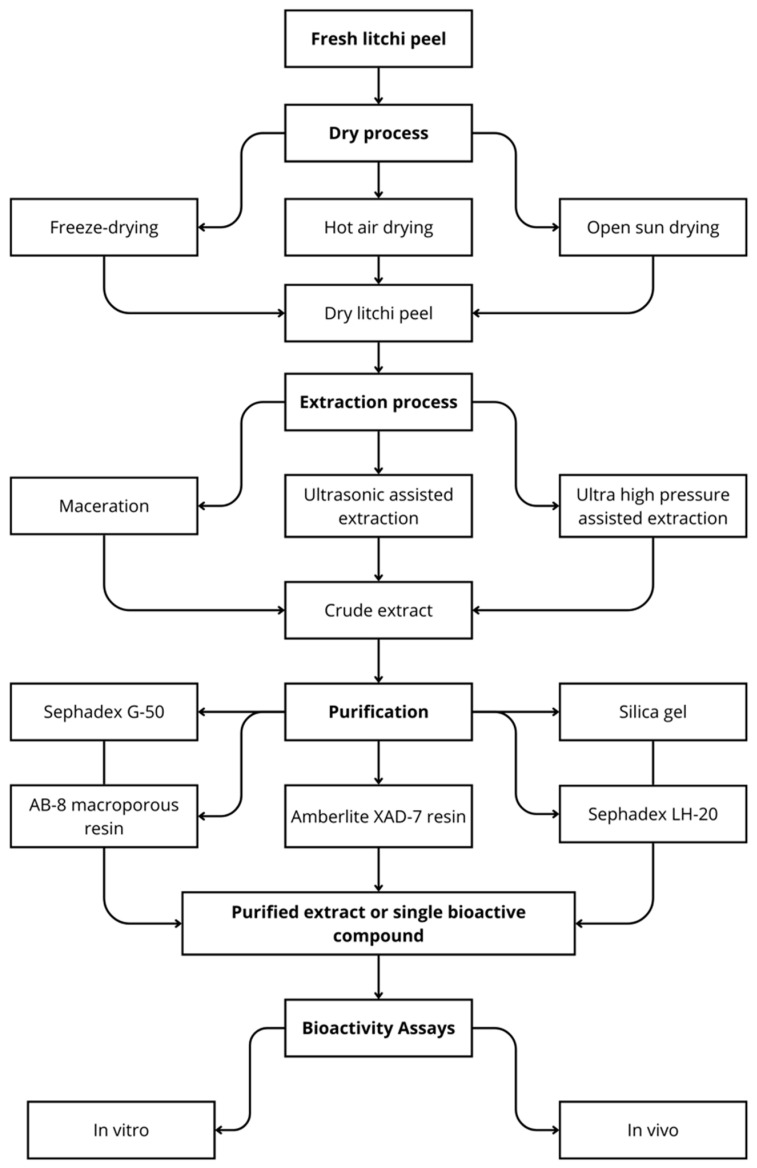
Overview of the study of litchi peel.

For the investigation of litchi peel, a sequential process must be undertaken, as depicted in Figure 1. This process starts with drying, followed by extraction, purification, and performed biological assays. The initial step we will discuss is the drying phase.

The drying process significantly affects the phytochemical compounds in fruits and vegetables. These phytochemicals, including phenols and flavonoids, are bioactive compounds found in plants with antioxidant abilities. These pharmacological properties can vary depending on drying, storage conditions, and storage time. Different drying techniques can induce positive and negative variations in the physical and chemical structure of dehydrated products. Litchi peels, a by-product frequently discarded, represent about 15% of the total weight of fresh litchi, causing significant waste of resources and environmental pollution. Some plant chemicals, such as flavonoids [10], proanthocyanidins, and anthocyanins, are increasingly known for their biological activities [14]. Litchi peel needs to be dried before its bioactive compounds are extracted. Drying methods can have an impact on the quality and quantity of plant chemicals extracted, hot air-drying, and freeze-drying have been used in the litchi peel process, as shown in Table 1.

### 2.1. Hot Air-Drying

The use of hot air-drying is necessary when rapid drying is required to reduce the cost of production. However, this technique often results in a significant loss of antioxidants due to the use of high temperatures. Different drying methods were applied to analyze their impact on the phytochemical profile and antioxidant activity of various products, and the results showed an increase and decrease in the content of bioactive compounds. Hot air-drying, conducted in controlled environments featuring ventilated spaces with varied heat sources, allows for precise temperature regulation and atmospheric pressure to optimize the drying process. The choice of drying technique for vegetables depends on their bioactive components. Using temperatures ranging from 100 °C to 180 °C enhances the drying process and is feasible for flavonoid-rich vegetables. However, applying such temperatures can result in the loss of volatile compounds [33,34].

The hot air-drying process involves a series of physical and physicochemical transformations in the plant material. The temperature of the drying medium, typically air, has a significant impact on the process. With increasing temperature, the drying rate is accelerated. It is essential to consider both the temperature and the duration of drying concerning bioactive compounds, which highlights the need to specify in detail the experimental conditions in any methodology employed [35,36]. According to the data in Table 1, different experiments use different oven-drying conditions and report variations in the bioactive composition of litchi peel. Babbar et al. [19] performed an 18-h oven-drying process at 60 °C and achieved a total phenol content of 3.68 mg GAE/g-PS. Similarly, Miranda-Hernandez et al. [27] used the same 60 °C drying method, but extended it to 48 h, reporting 2.2% procyanidin content (methanol) and 6.9% (acetone). On the contrary, Duan et al. [22] decided to dry at a lower temperature of 28 °C, but recorded anthocyanin content of 18.6 mg/100 g in a short time. Kessy et al. [23] investigated the influence of steam bleaching and drying process on phenolic compounds in litchi peel. To date, this is the only work that contrasts different drying parameters using varied methods for litchi peel. The samples were subjected to treatments that included steam bleaching for 3 min, followed by convection oven-drying at 60 and 80 °C, ambient drying, and convection oven-drying at temperatures 40, 60, 70, and 80 °C. Total phenolic compounds, flavonoids, proanthocyanidins, and anthocyanins decreased by 26.44%, 15.11%, 14.12%, and 78.61%, respectively, after ambient dehydration for seven days. Oven dehydration at 40 °C reduced total phenols, flavonoids, and anthocyanidins by 12.03%, 18.38%, and 60.01%, respectively. However, when the temperature exceeds 60 °C, there is a decrease in phenolic compounds that exceeds 40%. Combining steam bleaching and 60 °C oven-drying can maximize the concentration of phenolic compounds in litchi peel. The effect of hot air-drying on the phytochemical profile and antioxidant activity varies between fruit peels.

A study found that hot air-drying decreased the total phenolic content and antioxidant capacity of citrus peels, suggesting a potential degradation of bioactive compounds at higher drying temperatures [37]. Similarly, sun-drying decreased the antioxidant activity and betalain contents in dragonfruit peel, whereas hot air-drying at a controlled temperature preserved the betalain content in dragonfruit peel [38]. In contrast, a study on carrot peel showed that hot air-drying at typical temperatures resulted in lower levels of phytochemical compounds when compared to other methods like microwave-drying [39]. Research conducted on Hongjv (*Citrus reticulata Blanco*) peel noted that hot air-drying decreased the total polyphenol content and antioxidant activity [40]. The comparison of these findings with those found in litchi peel reveals that litchi peel can retain certain bioactive compounds post-hot air-drying, whereas other fruit peels may experience a reduction in antioxidant metabolites. This suggests that a drying technique should be selected considering the specific type of fruit peel and the desired outcome for bioactive compound preservation. The different results obtained with this methodology further reinforce the idea that the selected methods can significantly affect the phytochemical profile of dried products, underlining the importance of careful consideration and adjustment of these parameters.

### 2.2. Freeze-Drying

Freeze-drying is an advanced dehydration procedure that operates under low temperatures, thus ensuring optimal preservation of product integrity and quality. This technique, known as sublimation drying, focuses on removing moisture from previously frozen material by transitioning directly from a solid to a gaseous state, without passing through a liquid phase. The start of the process is crucial and consists of rapid freezing between −40 °C and −50 °C to prevent the generation of large ice crystals. This step, involving sublimation, utilizes a vacuum combined with a controlled temperature and can last up to 48 h. It follows the initial freezing stage, where the material is solidified for moisture removal. During this period, the moisture does not liquidize but volatilizes. Finally, a desorption stage is carried out at temperatures of 40–50 °C to remove the retention of non-frozen water. The resulting product retains only 1–2% of its original aqueous content [10,35].

In contrast to conventional drying methods, freeze-drying is renowned for its distinctive advantages. Standard techniques can induce chemical or physical alterations in products due to the high temperatures used, which, in the case of sensitive botanical extracts, can seriously compromise the quality of the resulting extract. Some of the most notable contrasts between the two methods include the moisture removal technique, the percentage of water removed (with freeze-drying removing almost 98% of the water, in contrast to 70–80% in conventional methods), the durability of the product (freeze-dried products can keep for up to 20–30 years, whereas conventionally dried ones have a shelf life of 1–5 years), and the nutritional content (freeze-dried items retain a higher amount of vitamins and nutrients compared to those dried by conventional methods) [10,35]. According to the information contained in Table 1, several investigations have adopted this methodology using litchi peel. Li et al. [17] applied freeze-drying as a preparatory step in the extraction process, achieving a procyanidin content of 99.56 ± 1.2%. This result is comparable to that obtained by Sui et al. [16], who reported a procyanidin content of 95.85 ± 4.44%. In contrast, Chen et al. [6] reported a phenolic content of 81.4%. To date, no studies are available directly comparing drying methods and their influence on the extraction of phytochemicals from litchi peel. Freeze-drying has proven to be an efficient method in sample preparation for phytochemical extraction, evidenced by the high percentages of procyanidins and phenols obtained in the cited investigations. However, there is a gap in the scientific literature on the direct comparison of different drying techniques and their impact on the extraction of phytochemicals from litchi peel, highlighting the need for future research in this area. Freeze-drying has been extensively used to preserve the phytochemical integrity and antioxidant activity of fruit peels, including litchi peels. Freeze-drying maintains the quantity of bioactive compounds in many fruit peels. The freeze-dried peels of purple star apple, yellow cashew, and red cashew represented a considerable source of antioxidant compounds, indicating the method’s effectiveness in preserving antioxidants [41]. Another study showed that freeze-drying retained the antioxidant activities in apple pomace powder (APP) and pomegranate peel powder (PPP), thus confirming its role in preserving the antioxidant potential [42]. Compared to other drying methods, freeze-drying has shown better preservation of phenolic compounds and antioxidant activities in several fruit peels, including unmatured citrus fruits [43] and dragonfruit peels [38]. However, other studies suggest that freeze-drying generally ensures the retention of bioactive compounds, but a loss of antioxidant activity can occur in fresh fruit, as evidenced in research on tropical fruits like starfruit and mango [44].

Overall, the findings across different fruit peels corroborate the effectiveness of freeze-drying in maintaining phytochemical profiles similar to what is observed in litchi peel, affirming the applicability of this method for high-quality extract preparation. The choice of drying method is crucial to optimize the subsequent extraction of phytochemicals from litchi peel. The drying method should be selected considering the bioactive compounds and the extraction method.

### 2.3. Open Sun-Drying

The use of solar energy has historically been fundamental in food processing and storage practices, particularly in the dehydration of agricultural products. The use of solar radiation to dry food and crops represents a cost-effective and sustainable technique, contributing significantly to the economic improvement of small producers and rural communities. Despite its long tradition, natural sun-drying has presented challenges in terms of the quality and purity of the final product. This ancestral way of preserving raw materials has often culminated in inferior quality products and susceptibility to contamination. Although solar drying is still practiced, many have opted to abandon it due to the prolonged duration of the process and lack of control over dehydration variables, which directly impact the quality of the dried product [10,35,45]. In this context, Jiang et al. [26] employed sun-drying as a preparatory method for their study, noting that antioxidant activity increased with increasing concentration for each sample (10–72%), suggesting that despite its drawbacks, controlled sun-drying can still contribute positively to the retention of beneficial compounds in the dehydration process. For instance, using a heat pump or fluidized bed dryer maintained to a better extent the antioxidant activity and betalain content in dragonfruit peel, when compared to spending more time on sun-drying technique [38]. Another study on different parts of wild pomegranate fruits, including the peel, suggested that natural sun-drying was less effective in preserving total phenols and antioxidant properties compared to other drying methods such as solar tunnel drying or mechanical cabinet-drying [46]. Similarly, the sun-exposed peel of apple fruit exhibited different thermal dissipation and antioxidant systems compared to the shaded peel, demonstrating that natural factors such as light exposure within the tree canopy could influence the antioxidant properties [47].

These findings agree with observations made for litchi peel, where open sun-drying has been employed with an acknowledgment of its limitations in terms of control over the drying variables and the quality of the product. Jiang et al. [26] showed no difference in antioxidant activity in sun-dried litchi peel samples. Therefore, it is apparent that more controlled drying methods might offer better preservation of phytochemicals and antioxidants in fruit peels, including litchi.

## 3. Extraction Methods from Litchi Peel

The extraction efficiency of bioactive compounds in a plant depends on the extraction technique used. The procedure for obtaining phenolic compounds from plants becomes the determining step in related research. The extraction efficiency of bioactive compounds in a plant depends on the extraction technique used. Previous research has addressed extraction methods used on litchi peels, including traditional and emerging techniques. Traditional approaches include maceration and thermal extraction, which involves refluxing, stirring, and mixing. The scientific literature has often relied on these traditional techniques to obtain litchi extracts. However, maceration and thermal extraction can be time-consuming and resource-intensive, with a consequent impact on the efficiency and quality of the final product. With technological advancement, there has been a growing interest in implementing emerging extraction technologies, such as ultrasound-assisted extraction (UAE) and ultra-high-pressure extraction, to enhance the quality of extracts obtained from litchi peels. Aspects that commonly determine extraction efficiency include the characteristics of the substrate, the solvent selected, parameters such as temperature and duration of extraction, the ratio of liquid to solid, and the particle size of the sample. The solvent selection is crucial to the extraction effectiveness of the bioactive compounds. The solvent polarity directly affects the solubility of phenolic compounds [48,49,50,51]. Different concentrations and polarities of solvents were used to extract plant chemicals from litchi peel, as shown in Table 1.

### 3.1. Maceration

Maceration allows for the extraction of a wide array of phenolic compounds due to the polar nature of these substances, which is influenced by their carbon structure and substituents. The solvents used to extract compounds from litchi peel mainly include ethanol, acetone, methanol, and HCl at different concentrations, and ethanol and ethanol allowed for the extraction of phenol compounds from the litchi peel, whereas other studies used 70% ethanol to extract procyanidins from litchi peel, achieving procyanidin contents of 99.56 ± 1.2% (*w*/*w*) and 95.85 ± 4.44% (*w*/*w*) compared with grape seed procyanidins [16,17], respectively, whereas Miranda-Hernández et al. [27] used methanol to extract procyanidins from litchi peel, achieving procyanidin contents of only 2.2% (*w*/*w*), which might be due to the solubility of phenolic compounds in ethanol, where the polarity of phenolic compounds relies on the carbon structure and the nature and number of substituents [51]. In addition to influencing the efficiency of the extraction process, other parameters include extraction temperature, time, and liquid–solid ratio. As detailed in Table 1, the extraction temperatures range from 25 to 59 °C, the time ranges from 15 min to 12 h, and the liquid-to-solid ratio ranges from 1:5 to 1:20, based on the above data on litchi peel extraction. Proper optimization of these factors is essential to maximize extraction efficiency.

### 3.2. Ultrasonic Assisted Extraction (UAE)

UAE is a promising method, recognized for its cost-effectiveness and efficiency, featuring notably shorter extraction durations. This technique uses ultrasonic baths that vary in solvent concentrations, liquid-to-solid ratios, duration, temperature, and ultrasonic frequencies. These parameters are subject to change and require optimization based on the type of material. The ideal conditions, which include the types and concentrations of solvents, duration, and temperature to extract phenols from litchi peel using UAE, are increasingly becoming a subject of interest [51].

Prasad et al. [31] comparatively explored the performance of conventional and ultrasound-assisted extraction in obtaining flavonoids from litchi peels. Although the yields of crude extract obtained by UAE and conventional extraction were 24% and 1.83%, respectively, both methods found the total phenol content and antioxidant activity to be similar at equivalent concentrations. Rao et al. [30] evaluated the impact of ultrasonic strength, duration, and concentration of ethanol on the yield of cyanidin-3-rutinoside. Ethanol concentration is the most important factor influencing extraction efficiency [28]. In the different cultivars, the phenol composition showed a significant difference, with flavonoids, proanthocyanidins, and phenolic acids being the predominant phenol compounds, yielding a total concentration of soluble phenol in litchi peel of 51 to 102 g/kg^−1^ DW. Li et al. [29] evaluated the effect of combined enzyme/ultrasonic therapy on extraction yield, obtaining a yield of up to 13.5%, representing a six-fold increase compared to traditional ethanol extraction. Additionally, the study noted a greater abundance of oligomers, suggesting that ultrasonic efforts could facilitate the transformation of flavanol compounds during the extraction process. Other works optimized ultrasound-assisted extraction using a Box–Behnken experimental design by evaluating various parameters such as concentration, extraction time, and power [29,30]. Response surface plots revealed that ultrasonic power, time, and concentration markedly influenced the extraction yield of the cyanidin 3-rutinoside and oligomeric procyanidin content, reaching efficiencies of up to 89.6%. Li et al. [29] explored enzyme treatment-assisted extraction of litchi pericarp procyanidins (LPOPC) and detailed a two-step method involving enzymatic pretreatment followed by UAE, where variables such as ultrasound power, time, and liquid-to-material ratio were optimized using response surface methodology (RSM). The results demonstrated that the combined enzyme/UAE process notably improved the yield of LPOPC, ranging from 8.25% to 14.20%, compared to the yield of less than 9% with only enzymatic treatment. These findings suggest that extractive process optimization can be a valuable and effective tool for obtaining bioactive components from several plant matrices; however, the need for further research on process optimization in several extraction methods persists. This lack limits the ability to make effective comparisons between the extraction techniques that have been optimized.

UAE is notable for its cost-effectiveness and efficiency, with reduced extraction durations compared to traditional methods. The technique’s versatility is evident in its use of ultrasonic baths, which can be adjusted considering solvent concentrations, liquid-to-solid ratios, duration, temperature, and ultrasonic frequency, tailored to the specific type of extracted material. Optimization of these parameters is crucial to achieve ideal extraction conditions, particularly for the extraction of phenolic compounds from litchi peel. These advanced extraction techniques, particularly UAE, represent a promising direction for the efficient and effective extraction of valuable compounds from natural products, offering significant improvements over traditional methods in yield and quality.

### 3.3. High-Pressure Extraction (HPE)

HPE is among the recent and innovative methods for extracting bioactive compounds from natural materials. This technique involves applying high pressure to stimulate various phenomena, such as phase transitions, reaction dynamics changes, and alterations in molecular structure. These factors can induce reactions that generally favor volume reduction, enhancing the efficiency of the extraction process. This approach has gained attention for effectively isolating bioactive compounds, making an addition to extraction technologies [52].

Prasad et al. [31] conducted comparative experiments to investigate the effects of conventional extraction, UAE, and HPE on the efficiency of flavonoid extraction from litchi peel. Their findings indicated varying yields for crude extract: 1.83% for conventional extraction, 24% for ultrasound-assisted extraction, and 30% for HPE. A study established optimal conditions for the HPE of proanthocyanidins from litchi peel. Using a response surface analysis method with four factors at three levels, they determined the ideal conditions to be 295 MPa pressure, a 13-min pressure maintenance time, a liquid-to-solid ratio of 16.0 mL/g, and an ethanol concentration of 70% [32]. These studies highlight the diverse and evolving approaches to enhance the extraction of valuable compounds from natural sources.

The efficient extraction of bioactive compounds from plant materials such as litchi peels depends on the extraction method used. Key factors such as the choice of solvent, substrate characteristics, extraction temperature and duration, liquid-to-solid ratio, and particle size of the sample play a crucial role in the extraction efficacy. The polarity of the solvent is particularly significant, directly impacting the solubility of phenolic compounds.

Solvents in different concentrations, such as ethanol, acetone, methanol, and hydrochloric acid have proven effective in extracting compounds from litchi peels. Optimizing parameters such as temperature, extraction time, and liquid-to-solid ratio is essential for maximizing extraction efficiency. Moreover, emerging extraction techniques such as UAE and HPE have been developed and evaluated. These techniques have shown promise in efficiency and effectiveness, improving yields and quality of extracts compared to traditional methods such as maceration and thermal extraction, which, although widely used, can be more time- and resource-intensive.

Optimizing the extraction process, including the appropriate selection of solvents, and implementing advanced technologies, such as UAE and HPE, represent a valuable and effective direction for extracting valuable compounds from natural products, offering significant improvements over traditional methods in yield and quality.

## 4. Isolation and Characterization of Bioactive Compounds in Litchi Peel

Chromatography is an analytical technique that separates distinct components within a chemical mixture. This separation process hinges on moving a sample through a mobile phase that continuously encounters a stationary phase that remains fixed and sample-free. The categorization of chromatographic separation hinges on the physical states of the mobile and stationary phases, how these phases interact, and the chemical or physical mechanisms driving the separation of the sample’s components. This versatile method accommodates various separation techniques, including adsorption, partition, ion exchange, size exclusion, and electrophoretic separation. Each analytical technique is specifically chosen based on the solutes’ characteristics and the objectives of the purification endeavor [12]. Table 1 shows some works that include the purification of isolated bioactive compounds.

The quest to discover bioactive compounds from plant sources begins with extracting a mixture composed of diverse phytochemicals. This initial extract, rich in potential, is also laden with a myriad of compounds that can range from beneficial to inert, or even deleterious, necessitating a critical step of purification. The purification process is not merely a procedural necessity; it is a scientific imperative that ensures the isolation of specific compounds of interest, enabling a detailed study of their properties, mechanisms of action, and potential applications. Given the spectrum of phytochemicals present in a single extract, the task of purification is like navigating a labyrinthine world of molecular diversity. This endeavor is crucial for eliminating interferences, concentrating desired molecules, and facilitating a precise characterization of bioactive constituents. The intricate world of phytochemical research, particularly in flavonoid derivatives, is deeply enriched by the diverse array of chromatographic techniques developed and refined over time. Traditional and more contemporary approaches play a role in isolating, identifying, and characterizing compounds extracted from various plant sources.

At the forefront of these chromatographic methods is size-exclusion chromatography, a type of partition chromatography that has been instrumental in isolating molecules based on their molecular sizes. Referred to by various names, including gel-permeation, gel-exclusion, gel filtration, and molecular-sieve chromatography, this technique is renowned for its efficacy in size-based separations. Separation in size-exclusion chromatography is achieved through the interaction of molecules with porous matrix column packing, resulting in differential degrees of access. Molecules smaller than or equivalent in size to pores can penetrate the matrix, whereas larger molecules are excluded, facilitating their separation. The success of this method hinges on critical parameters such as the diameter and pore size of the packed materials, the choice of the appropriate eluent as the mobile phase, and the length of the column used. The versatility and effectiveness of size-exclusion chromatography make it a standout method among the most used techniques for size-based separations in phytochemical research [12,53].

### 4.1. Silica Gel

The utilization of polyamide in conjunction with silica gel chromatography significantly augments the efficiency and specificity of the isolation and characterization process for various compounds, especially flavonoids, polyamide’s intrinsic property to form hydrogen bonds with the hydroxyl groups of flavonoids emerges as a pivotal factor in the chromatographic separation process. This interaction is beneficial in enhancing the selectivity and resolution of flavonoid compounds, where the number and positions of hydroxyl groups critically influence their isolation and purification. By leveraging the hydrogen bonding capability of polyamide, chromatographic fillings can achieve a more refined separation, especially for high-polarity flavonoids, including glycosylated forms, which are efficiently separated using C18 silica gel in both normal and reverse-phase chromatography. This synergy between polyamide and silica gel chromatography broadens the scope of separable compounds and elevates the method’s overall efficacy, making it a cornerstone technique in the chromatographic separation of compounds with varied polarities [12,13].

### 4.2. Sephadex LH-20 (SLH)

SLH, a fundamental tool for purifying phytochemicals, has been widely used in various plant families for isolating diverse flavonoids and flavonoid derivatives. SLH has been instrumental in isolating 189 documented flavonoids, primarily from the Asteraceae, Moraceae, and Poaceae families. Its effectiveness is underscored by the successful isolation of 79 flavonols, 63 flavones, and 18 flavanones. Furthermore, SLH has facilitated unique isolations of homoisoflavanoids and proanthocyanidins from the Asparagaceae and Lauraceae families, respectively, with the Asteraceae family emerging as a rich source of flavones, boasting 22 distinct derivatives. The continued reliance on the success of SLH in phytochemical purification is attributed to its cost-effectiveness, convenience, rapidity, and efficiency, making SLH an indispensable tool in the analytical field [12,13]. SLH presents high efficiency in the purification of a range of substances, with some procedures achieving purities above 90% and total yields exceeding 40% [54].

Some advantages of using SLH include its high efficiency in separating plasma corticosteroids, antibodies, and other biological substances. The columns can be reused many times after appropriate washing, offering economic and practical benefits. Additionally, the system provides good results at temperatures below 25 °C, underscoring its suitability in a wide range of laboratory settings [55]. However, there are disadvantages to consider. SLH chromatography can be challenging to automate due to the need for a constant flow rate, which is difficult to maintain with gravity flow. Compatibility with materials also poses a problem, as solvents can only encounter certain materials like glass, stainless steel, and Teflon. Another issue is the low surface tension of the eluents, which can prevent the efficient splitting of the eluate stream for fraction collection, an important consideration when designing experimental protocols [56].

In conjunction with these methods, affinity chromatography has been recognized as an effective strategy for the selective isolation of specific proteins from crude extracts. The development of affinity matrices, which was historically a time-consuming and laborious task, has been significantly streamlined through the application of molecular biology techniques. This innovation encompasses the molecular integration of the capture molecule with an agarose-binding domain (ABD), effectively merging the steps of purification and attachment into a single, efficient process. Matrices based on dextran, like Sephadex, together with agarose, have become widely acknowledged for their use in bio-affinity chromatography. The straightforwardness, effectiveness, and swift preparation of the affinity matrix by employing fusion proteins produced recombinantly straight from crude cell extracts render affinity chromatography a preferred method for the purification of many proteins [57,58].

### 4.3. Amberlite XAD-7

Zhang et al. [18] observed a groundbreaking approach in litchi anthocyanin studies. Amberlite XAD-7 resin column demonstrated a pronounced affinity towards isolating anthocyanins from litchi, effectively segregating them from salts, carbohydrates, and other soluble substances. This separation identified a primary anthocyanin fraction, distinguished by its maximum absorption at 510 nm. Subsequent refinement using an SLH column, which differentiates based on molecular size, yielded four distinct anthocyanin fractions with varying absorbance levels at the same wavelength, indicating a comprehensive purification process validated for anthocyanin isolation in this context. The anthocyanin fraction, isolated through the SLH column, was further analyzed using HPLC-MS, revealing a significant peak indicative of a high-purity compound. The mass chromatography profile pinpointed the molecular weight of the leading anthocyanin in the litchi peel at 595 *m*/*z*, assumed to be cyanidin-3-rutinoside according to previous assumptions. This analysis was bolstered by electrospray mass spectrometry, which identified two components within the *m*/*z* 595 fraction: one correlating with cyanidin-3-glucoside at an ion peak molecular weight of 449, and the other aligning with cyanidin at an ion peak molecular weight of 287 g/mol. The ability of Amberlite XAD-7 resin and SLH columns to effectively isolate and purify anthocyanin fractions from litchi peel, coupled with advanced analytical techniques like HPLC-MS, highlights the precision and efficacy of these methodologies in uncovering the nuanced composition of phytochemicals. Gong et al. [11] isolated, purified, and concentrated anthocyanins effectively from litchi extract, employing a two-stage chromatographic process and using Amberlite XAD-7 resin and SLH columns, which facilitated the segregation of extracts into three primary fractions according to their anthocyanin content. Subsequent analysis of further partitioned fractions (P1–P4) through LC-MS and HPLC techniques revealed the presence of several key compounds. The most notable compounds identified were epicatechin in fraction P1, a prominent phenolic compound known for its antioxidant properties, and a B-type procyanidin dimer in P2, indicative of the intricate polyphenolic composition of the litchi peel. Furthermore, fractions P3 and P4 produced proanthocyanidin trimers, including both B2A and B types, along with A-type dimers and a unique trimer featuring an afzelechin or epiafzelechin unit, further showcasing the diversity of procyanidins in the litchi peel. These findings not only enhance our understanding of the phytochemical profile of litchi peel but also underscore the potential of these bioactive compounds to contribute to the fruit’s health-promoting properties. Amberlite XAD-7 resin has proven to be highly effective in isolating anthocyanins from litchi, with purification yields exceeding 90% in some applications, demonstrating its efficacy in achieving high-purity compounds [59]. Despite its high yield and effectiveness, Amberlite XAD-7 faces challenges in automation due to fluctuations in flow rate with gravity flow, and material compatibility issues, as only certain materials like glass, stainless steel, and Teflon should contact the solvents. Additionally, the low surface tension of the eluents may complicate the splitting of the eluate stream [56].

### 4.4. Sephadex G-50

Yang et al. [25] added a new dimension to this field by purifying polysaccharides from litchi. The DEAE anion exchange chromatography method coupled with the anion exchange column facilitated the division of these polysaccharides into two distinct peaks, named F1 and F2, with the F1 fraction predominating at 82% of the total polysaccharide yield, demonstrating a high purity level of 99.5%. The subsequent step involved the application of gel filtration on Sephadex G-50, which further refined the F1 fraction into a singular, highly purified fraction F01, marked by a specific retention time. This purification process was instrumental in achieving a fraction (F01) with an exceptionally high polysaccharide content of 99.9%, underscoring the effectiveness of the chromatographic techniques. However, this method presents some disadvantages. Sephadex G-50 can be associated with issues related to flow rates, especially when using gravity flow, which can be challenging to maintain consistently. This type of gel filtration chromatography often requires careful choice of buffers and may involve longer preparation times compared to other chromatographic techniques. Despite these challenges, the advantages of Sephadex G-50, such as its ability to effectively separate components based on size with high reproducibility and without significant loss of material, make it a valuable method in biochemical analysis. This balance of high yield and effective purification against practical considerations highlights the ongoing relevance of Sephadex G-50 in complex purification processes [60]. Jiang et al. [26] employed an elaborate and multi-step purification process to isolate various compounds from litchi. Beginning with a reddish solid obtained from the extraction solution, they utilized a sequence of solvent fractionations, followed by silica gel column chromatography. This meticulous approach involved a chloroform-methanol solvent system of varying polarity and yielded ten distinct fractions. Eight compounds were isolated and identified, each with distinct structures and chemical properties. A comprehensive analysis was performed by mass spectrometry (ESI-MS) and nuclear magnetic resonance spectroscopy (NMR). This analytical approach allowed for precise identification of the compounds from their molecular ions and spectroscopic data, with comparisons with the existing literature to confirm their identities. The identified compounds included methyl 3,4-dihydroxybenzoate, a compound with three aromatic protons and a specific molecular ion, and stigmasterol, a colorless crystal characterized by its unique NMR spectra and the presence of olefinic protons. Isolariciresinol was noted for its crystalline form and the detection of aromatic methoxyl and methylene groups related to hydroxyl groups, indicative of its complex structure. Kaempferol was identified through its NMR spectrum, which includes six aromatic protons and a distinctive carbonyl signal. A novel natural compound, described by its ortho-substituted benzene ring and a methoxy group signal, was identified, showcasing the diversity of compounds in the litchi peel. Methyl shikimate and ethyl shikimate were characterized by their AB spin system and the presence of specific hydrogen and carbon signals, respectively, demonstrating variations in their chemical structures. Butylated hydroxytoluene, a synthesized antioxidant, was also identified, suggesting its natural occurrence as an antioxidant in the litchi peel. This selection of purified compounds underscores the chemical diversity and potential biological significance of the constituents of the litchi peel. Flavonoids and steroids with antioxidant activity highlight the intricate chemical makeup of litchi peel and its potential as a source of bioactive compounds.

### 4.5. AB-8 Macroporous Resin

The work of Sui et al. [16] brought a fresh perspective to the table, focusing on the purification of procyanidins from litchi peel. Their innovative approach began applying a crude aqueous procyanidin solution to a column packed with AB-8 macroporous resin. The process included several stages of rinse and elution for the obtention of litchi peel procyanidins (LPPC). Their findings, particularly the high content of procyanidins in LPPC, underscored the potential of litchi peels as a novel source of A-type procyanidins. The analysis of procyanidins within litchi peel was performed by assessing the peak areas (%) of thirteen identified peaks, thereby facilitating the quantification of the relative content of A-type and B-type procyanidins. The peak area percentages served as indicators of each compound’s relative proportion within the LPPC, utilizing the concept that a 100% relative abundance implies a predominant presence within a specific peak. The collective area of these 13 peaks constituted 89.44% of the total peak area of the LPPC, highlighting the significant presence of procyanidins. The monomeric constituents, (−) epicatechin and (+)-catechin, accounted for 37.92% and 4.10% of LPPC, respectively. A-type procyanidins, including dimers and trimers, formed 38.76% of LPPC, whereas B-type procyanidins constituted a lesser portion, at 8.66%, with A-type procyanidins approximately 4.48 times more abundant than B-type. This differential presence underscores the potential of litchi peels as a rich source of A-type procyanidins. Continuing this trend, Chen et al. [6] focused on the purification of polyphenols from litchi extract. Using AB-8 macroporous resin and various chromatographic techniques was crucial in removing impurities and isolating the desired compounds. The quantification of polyphenol content in the extracts also highlighted significant differences in phenolic content between different litchi peel extracts. UPLC-MS, ESI-MS, and NMR facilitated the structural identification of compounds, including procyanidin B2, (-)-epicatechin, an epicatechin complex derivative, an A-type procyanidin trimer, a B-type procyanidin dimer, and procyanidin A2. Among these compounds, epicatechin was the predominant monomer in litchi peel extract, whereas the content of the B-type procyanidin dimer was comparatively minimal. This study emphasized the importance of precise quantification and advanced chromatographic techniques to understand the composition of litchi polyphenol extracts. Despite achieving a high yield, which indicates the method’s efficacy in isolating high-purity compounds, using AB-8 macroporous resin presents disadvantages. For instance, AB-8 offers high adsorption capacity; however, the operation can be limited by issues such as material compatibility, as only certain materials like glass, stainless steel, and Teflon should contact the solvents. AB-8 may require specific conditions to optimize the elution and adsorption processes, which can add complexity to the purification process [61]. Nevertheless, the advantages of using AB-8 resin, such as its cost-effectiveness, high efficiency, and simplicity in setup and operation, make it a valuable tool in purificating complex biomolecules. This balance of high yield and effective purification against practical considerations demonstrates the ongoing relevance of AB-8 macroporous resin in complex purification processes [62].

The categorization of the elution into distinct fractions based on absorbance characteristics demonstrated a refined method for extracting these valuable compounds from litchi extracts, showcasing the nuanced approach needed for such tasks. The cumulative research from these studies presents a rich and detailed narrative of the purification processes applied to litchi extracts. Each study, with its unique focus and methodology, contributes to a broader understanding of the complexities involved in extracting and purifying compounds from natural sources. This body of work advances our knowledge in the natural product extraction field and lays the groundwork for future explorations into the potential applications of these purified compounds.

The convergence of multiple chromatographic techniques in the purification of phytochemicals underscores a significant evolution in phytochemical research. This evolution is characterized by a blend of traditional methods and modern innovations, each of which contributes uniquely to the extraction and purification processes. Key methodologies like size-exclusion chromatography, silica gel chromatography, and affinity chromatography have each played a pivotal role in advancing our understanding and capability to isolate specific compounds from litchi peel sources with high efficiency and precision. Size-exclusion chromatography, known for its effectiveness in separating molecules based on molecular size, emerges as a standout method in this field. Silica gel chromatography further enhances the landscape of phytochemical purification. This method’s ability to isolate and characterize compounds based on their polarity marks it as a robust and versatile tool in the chromatographer’s arsenal. The significant role of SLH in the purification of a wide range of polyphenols has been described. This work advances our knowledge in natural product extraction and sets the stage for future investigations into the potential applications of these purified compounds, cementing the crucial role of chromatographic techniques in phytochemical research. Litchi peel has emerged as a significant source of bioactive compounds, with studies revealing a diverse array of substances that have the potential for health-promoting applications. In particular, the isolation and identification of anthocyanins and procyanidins underscore the rich phytochemical diversity of litchi peel. The presence of compounds like epicatechin and kaempferol further enriches the profile of the litchi peel, suggesting avenues for exploring their roles in modulating biological processes and disease mechanisms.

## 5. Biological Properties and Mechanisms of Action of the Bioactive Compounds in Litchi Peel

**Figure 2:**
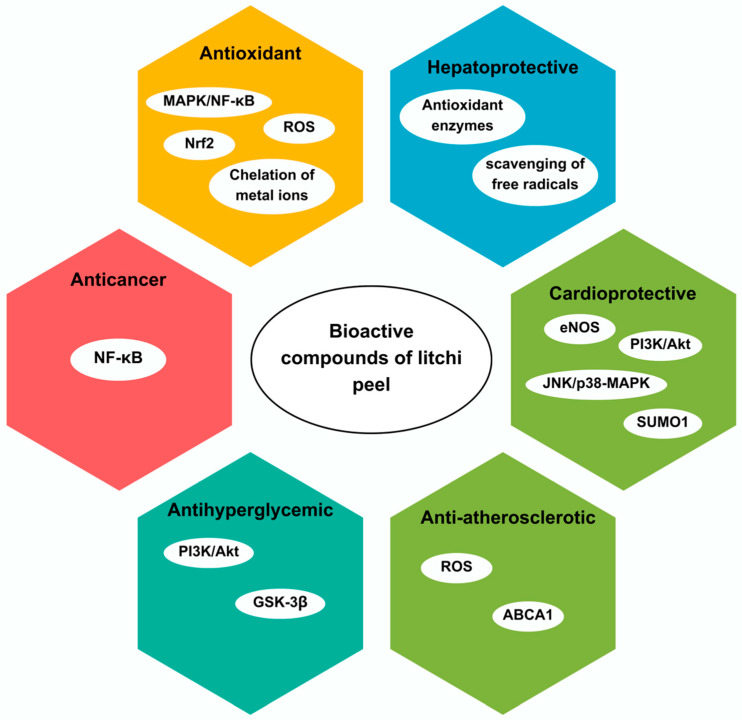
Mechanisms of health benefits mediated by bioactive compounds of litchi peel.

This section examines a selection of studies that explore the bioactivities and mechanisms of action of various bioactive compounds extracted from litchi peel (Figure 2), including antioxidant, antihyperglycemic, cardioprotective, hepatoprotective, anti-atherosclerotic, anticancer, and anticoccidial activities, in various *in vitro* and *in vivo* tests, as shown in Table 2.

### 5.1. Antioxidant Activity

Yang et al. [25] evaluated DPPH scavenging activity, indicative of antioxidant potential, utilizing a water-based extract from litchi peel polysaccharides. The extract underwent chromatography through an anion exchange column, yielding two separate fractions. The fraction containing the purified polysaccharides, tested at 100 µg, showed the highest antioxidant activity (54%). This result suggests that the purified polysaccharide from litchi peel might represent a novel antioxidant agent. Liu et al. [7] explored the hydroxyl radical (OH) scavenging capabilities of oligomeric proanthocyanidins (PCs) derived from litchi peel by testing various concentrations. Their findings revealed that all the concentrations assessed had a potent effect in scavenging hydroxyl radicals. The IC_50_ values, which represent the concentration needed to neutralize 50% of the free radicals, were determined to be 2.60 µg/mL for oligomeric PCs, 1.75 µg/mL for A2 PCs, and 1.65 µg/mL for trimeric PCs, showcasing the efficacy of these compounds in radical inhibition. The study also suggested that the antioxidant activities of dimeric and trimeric type-A PCs might be related to the number of hydroxyl groups in their molecular structures. Jiang et al. 2013 [26] highlighted the potent antioxidant properties of PCs and their potential applications in health and wellness. The study investigated the antioxidant properties of bioactive compounds isolated from litchi peel, such as kaempferol and isolariciresinol, which demonstrated notable antioxidant activities evidenced by their performance in free radical scavenging assays compared to butylated hydroxytoluene (BHT), a well-known synthetic antioxidant. The results of the DPPH assay revealed a concentration-dependent enhancement of antioxidant activity for each sample, indicating that the higher the concentration of the compounds, the more pronounced their antioxidant effects. In particular, the scavenging effects of kaempferol and isolariciresinol surpassed those of BHT, highlighting their potent antioxidant capabilities. The primary mechanism of antioxidant activity of kaempferol involves its ability to reduce the production of free radicals and reactive oxygen species (ROS). This action is crucial, as ROS are highly reactive molecules that can cause cellular damage, leading to various diseases, including cancer, cardiovascular diseases, and neurodegenerative disorders. Kaempferol achieves this reduction by directly scavenging free radicals, thus neutralizing their reactivity, and preventing them from causing oxidative stress within cells [64]. Yang et al. [14] evaluated the radical scavenging activity of litchi peel extract, as indicated by IC_50_ = 1.22 µg/mL in the 1,1-diphenyl-2-picrylhydrazyl (DPPH) assay and IC_50_ = 1.12 µg/mL in the 2,2’-azino-bis (3-ethylbenzothiazoline-6-sulfonic acid) (ABTS) assay. The study also examined the effects of D-galactose administration in mice, which is known to induce oxidative stress and mimic aging-related changes. Administration of 400 mg/kg D-galactose reduced antioxidant enzyme activities, exacerbated lipid peroxidation, and induced protein oxidation in mice. The levels of glutathione, superoxide dismutase, and malondialdehyde were restored, compared to the control group (mice without treatment), in aged mice after treatment with the litchi peel extract (400 mg/kg) for 8 weeks. This suggests that the extract has potential therapeutic properties against oxidative stress and age-related cellular damage, highlighting its potential as a natural antioxidant supplement. Polysaccharides have an interesting mechanism regarding their ability to prevent the generation of free radicals; this mechanism involves the chelation of metal ions, such as ferrous and copper ions, which play a pivotal role in the catalysis of the Fenton reaction. By binding to these transition metal ions, polysaccharides effectively inhibit the progression of the reaction, thus reducing the formation of these damaging radicals [63]. PCs exhibit a multifaceted mechanism of action in their antioxidative capacity, crucial to mitigating oxidative stress (OS)-associated diseases [65]. Their effectiveness spans both *in vitro* and *in vivo* assays by decreasing scavenging harmful molecules and modulating key signaling pathways that govern cellular responses to stress. PCs enhance the cellular antioxidant framework through several distinct mechanisms. One primary avenue through which PCs exert their antioxidant effects is scavenging hydroxyl radicals and superoxide anions. This direct interaction with reactive oxygen species (ROS) helps neutralize these harmful molecules before they can induce cellular damage. Beyond this direct scavenging activity, PCs significantly influence cell defense mechanisms [65] and activate the Nrf2 pathway, a critical regulator of the cellular antioxidant response, leading to the upregulation of various endogenous antioxidant enzymes and detoxification proteins. which enhances the inherent antioxidant capacity of the cell. PCs modulate other signaling pathways, notably inhibiting the MAPK/NF-κB pathway, involved in inflammatory responses and oxidative stress conditions. [65,72,73,74,75]. The multifaceted antioxidative mechanisms of PCs and polysaccharides, from direct radical scavenging to modulation of cellular antioxidant defenses and signaling pathways, underscore their potential as versatile and safe antioxidants in combating diseases related to oxidative stress; however, it is noteworthy that the evaluation of individual isolated compounds within the PCs spectrum remains largely unexplored. This research gap presents a pivotal opportunity for future studies to focus on the isolation and detailed examination of single PCs and polysaccharide compounds. Such targeted investigations are crucial for unraveling the specific mechanisms of action attributed to individual PCs and polysaccharides, thus enhancing our understanding of their therapeutic potential. Other studies on the antioxidant activity of fruit peels have been carried out. Babbar et al. [19] demonstrated that kinnow mandarin peel extract exhibited the highest antioxidant activities among several fruit residues; litchi pericarp also showed significant potential, indicating its antioxidant properties comparable to other fruits. Okonogi et al. [76] evaluated the antioxidant capacities of various fruit peels and found that certain peels like pomegranate and rambutan exhibited free radical-scavenging power. This study noted that litchi peel extract has beneficial antioxidant effects; some other fruit peels might offer stronger or comparable antioxidant activities.

### 5.2. Cardioprotective Activity

Chen et al. [15] obtained a purified extract from litchi peel. The main compound identified in this extract was B-type procyanidin dimer. The capacity of the pure extract to eliminate oxygen free radicals was assessed using *in vitro* assays (IC_50_ = 120 g/kg in the DPPH assay). The purified extract (200 mg/kg) restored the levels of lactate dehydrogenase, compared to the group without any treatment, and increased Bcl-2 expression and decreased Bax expression in myocardial tissue in a model of acute myocardial ischemia in Sprague–Dawley rats for 5 days.

The study by Ramirez-Sanchez [77] showed that epicatechin 1 µM exerted the highest inhibitory activity on NO production in human coronary artery endothelial cells at 10 min of treatment; such effects were through serine 633 and serine 1177 phosphorylation and threonine 495 dephosphorylation in endothelial nitric oxide synthase, an enzyme that produces nitric oxide (NO), a vasoprotective molecule that plays a fundamental role in maintaining vascular health by improving NO bioactivity and simultaneously reducing superoxide production [67]. Acute administration of epicatechin has been shown to induce eNOS activation in human coronary endothelial cells, leading to improved endothelial function and vascular health [67]. Beyond its effects on eNOS and NO bioactivity, epicatechin exerts cardioprotective effects through several other molecular mechanisms, especially in ischemia/reperfusion (I/R) injury. These mechanisms include inhibition of apoptosis and activation of cardioprotective signaling pathways such as the PI3K/Akt pathway, known as the RISK pathway [78]. Additionally, epicatechin can inhibit stress-associated signaling pathways, including JNK/p38-MAPK, contributing to the preservation of mitochondrial function and modulation of autophagy processes [67,78].

Furthermore, epicatechin showed a protective effect against cardiac fibrosis, a critical factor in the progression of heart disease. This effect is mediated through the regulation of SUMO1-dependent modulation of SIRT1, a pathway that further underscores the therapeutic potential of this compound in cardiac health [79]. Through these diverse mechanisms, epicatechin presents a promising natural compound for developing interventions aimed at cardiovascular protection and the mitigation of heart disease. Other studies were conducted with other fruit peels. Paul et al. [80] showed the cardioprotective effects of epicatechin, a compound found in several fruits, including litchi. Similarly, research on *Citrus macroptera* peel extract demonstrated significant cardioprotective effects against myocardial infarction in rats, suggesting that the peel of this fruit, like litchi, may have valuable cardioprotective properties. In addition, extracts of *Annona crassiflora* fruit peel exerted antioxidant properties in the cardiac tissue of hyperlipidemic mice, indicating a cardioprotective potential comparable to that shown by litchi peel extracts [81].

### 5.3. Hepatoprotective Activity

Chen et al. [6] extracted distinctive polyphenolic compounds from the litchi peel and performed *in vitro* and *in vivo* experiments to evaluate their liver-protective effects. The plant extract (100 µg/mL) increased the viability of carbon tetrachloride (CCl_4_)-injured BNL CL.2 liver cells by approximately 20%, and the plant extract tested at 200 mg/kg decreased the serum levels of aspartate aminotransferase and alanine aminotransferase, markers of liver damage, in CCl_4_-intoxicated male ICR mice after 6 weeks of treatment. The possible mechanism of this activity relied on restoring the glutathione antioxidative system (glutathione peroxidase and glutathione reductase) at levels of mice without any pharmacological treatment. The hepatoprotective activity was confirmed when the extract preserved the hexagonal structure of hepatocytes and decreased the number of necrotic cells, with histopathological examinations of the livers of ICR mice exposed to CCl_4_.

Similarly, Alkinani et al. [68] evidenced the hepatoprotective activity of epicatechin (20 mg/kg) for 3 weeks in (CCl_4_)-induced acute liver injury model in Wistar rats, restoring the levels of aspartate aminotransferase, alanine aminotransferase, and alkaline phosphatase compared to those found in control animals (without treatment). The regulation of antioxidant enzymes and the direct scavenging of free radicals by the compound itself collectively contribute to the mitigation of oxidative stress, a key factor in the pathogenesis of liver damage.

Feng et al. [71] showed that a flavonoid-rich extract of litchi peel (25 and 100 mg/kg) exerted hepatoprotective activity in a model of CCl_4_-induced liver fibrosis in rats by decreasing the serum levels of alanine aminotransferase and aspartate aminotransferase after 4 weeks of treatment. A molecular docking study revealed that procyanidin A2, a bioactive compound belonging to the procyanidin class, interacts with several molecular targets and signaling pathways critical to liver health and disease. The participation of the PI3K-Akt signaling pathway underscores a mechanism through which procyanidin A2 may exert protective effects against liver fibrosis, a disease characterized by excessive accumulation of extracellular matrix proteins leading to scarring of liver tissue. The HIF-1 (hypoxia-inducible factor 1) signaling pathway, involved in cellular responses to oxygen deprivation presented in chronic liver disease, is another mechanism through which procyanidin A2 might act. By modulating this pathway, procyanidin A2 could help mitigate hypoxia-induced tissue damage and fibrosis. Although procyanidin A2, along with other compounds such as pinocembrin, quercetin, epicatechin, naringenin, nobiletin, phlorizin, and rutin, has been identified to have a high correlation with liver fibrosis-related targets and pathways, it is imperative to note that conclusive evidence delineating the specific activity of procyanidin A2 has not yet been established. The current understanding does not isolate the hepatoprotective mechanism of action to procyanidin A2 alone, but rather to a synergistic effect of the group of compounds. This underscores the need for more studies to understand the specific contributions of procyanidin A2 to liver health. Other studies have been conducted on various fruit peels. Gad et al. [82] found that grapefruit and orange peel extracts at 200 mg/kg showed significant liver protection against lipopolysaccharide-induced liver damage in rats. These citrus peels showed similar or even better results compared to standard treatments, indicating their potential as effective natural treatments for liver conditions. Similarly, passion fruit peels demonstrated hepatoprotective activity against acetaminophen-induced liver toxicity in rats, suggesting that these extracts may also protect against liver damage [83]. Both purple and red passion fruit peels effectively reduced liver enzyme levels, comparable to those of the standard drug silymarin, underscoring their significant potential.

### 5.4. Anti-Atherosclerotic Activity

Rong et al. [9] explored the effects of procyanidins extracted from litchi peel in combating atherosclerosis and hyperlipidemia. Their study focused on apolipoprotein E knockout (ApoE KO) C57BL/6 mice subjected to a high-fat diet (comprising 21% fat and 0.15% cholesterol). The findings revealed that treatment with litchi peel procyanidins (LPPC) (100 mg/kg) mitigated the development of atherosclerosis, diminished fat deposition, and ameliorated hyperlipidemia in the ApoE KO mice for 24 weeks. These results propose that procyanidins from litchi peel hold potential as a means for managing or preventing cardiovascular conditions like atherosclerosis and hyperlipidemia (decreasing the serum levels of cholesterol and LDL), in scenarios involving the consumption of high-fat diets by decreasing the mRNA levels of 3-hydroxy-3-Methylglutaryl (HMG)-CoA reductase, a mediator of cholesterol biosynthesis.

Queiroz et al. [8] examined the effect of litchi peel flour (LPF) on a range of health indicators associated with obesity, such as serum levels of total cholesterol (TC), low-density lipoprotein cholesterol (LDL-c), triacylglycerols (TAG), and other metrics linked to obesity in rats fed a hypercholesterolemic diet. LPF (5–10% in the diet) consumption inhibited body weight gain and body mass index, alongside lower levels of glucose, TAG, TC, LDL-c, liver enzymes, and leptin after 21 days of treatment. LPF decreased the percentage of hepatic lipids, hepatic lipid peroxidation, and instances of severe steatosis. Histological examinations of the aorta showed an absence of atheromatous plaque formation. These outcomes highlight the potential of litchi peel flour as a beneficial dietary element in managing obesity-related health issues and minimizing the risk of atherosclerosis.

Wang et al. [69] delved into the impact of PCs on atherosclerosis, PC administration (50 mg/kg) reduced, compared to control groups, atherosclerotic markers (LDL, triglycerides, and total cholesterol) in rabbits fed a 0.3% cholesterol diet after 24 weeks of treatment. The results revealed a notable decrease in oxidative stress marker staining alongside an increase in ATP-binding cassette subfamily A member 1 staining and expression at both mRNA and protein levels. ABCA1 plays a crucial role in cholesterol efflux and HDL formation, implying that PCs exert their anti-atherosclerotic effects by enhancing the body’s natural cholesterol regulation mechanisms and mitigating oxidative stress.

Zhang et al. [66] assessed procyanidin B2, along with other compounds, using network pharmacology analysis to elucidate its mechanism of action. This analysis revealed the compound’s engagement with signaling pathways and biological processes related to cardiovascular disease, as identified through KEGG mapping and Uniprot analysis. Procyanidin B2 alleviated endothelial cell damage, reduced levels of reactive oxygen species (ROS), and decreased foam cell formation in a dose-dependent manner, suggesting that procyanidin B2’s protective effects against atherosclerosis are mediated by the inhibition of endothelial cell damage and the reduction of oxidative stress. Together, these studies illuminate the various mechanisms through which procyanidins, including procyanidin B2, contribute to the prevention and regression of atherosclerosis. Other anti-atherosclerotic effects have been conducted with other fruit peels. Pomegranate peel extracts reduced atherosclerotic lesions and improved lipid profiles in apolipoprotein E-deficient mice, with similar activity to the results of studies on lychee peel [84]. In addition, grapefruit and kinnow mandarin peels have shown antioxidant activities related to anti-atherosclerotic effects due to their ability to reduce oxidative stress, a contributor to atherosclerosis [19].

### 5.5. Anticancer Activity

Gong et al. [11] utilized bioassay-guided fractionation through column chromatography to isolate active phenolic compounds from the aqueous extract of litchi peel. Their research found that a specific fraction (30 μg/mL) inhibited by 32.9% the viability of A549 cells at 48 h of treatment, showcasing higher effectiveness than other tested fractions and even surpassing the efficacy of platinum cis-dichlorodiamine (DDP) at a concentration of 0.5 μg/mL. However, this level of inhibition did not reach the effectiveness observed when all four fractions were combined. This study identified, for the first time, two types of B epicatechin trimers in the litchi species. The identified proanthocyanidins (PACs) were detected in the peel of the young fruit, highlighting the potential of these compounds in biomedical applications, especially cancer research. The mechanisms through which PACs exert their anticancer effects are multifaceted, involving intricate modulations of cellular signaling pathways. A pivotal aspect of PACs’ anticancer action is their impact on the NF-κB signaling pathway, PACs downregulated the lipopolysaccharide (LPS)-induced phosphorylation of p65, and the inhibitor of nuclear factor kappa B (IκB), which are crucial components of the NF-κB signaling cascade [85,86]. This downregulation impedes p65 translocation of p65 from the cytoplasm to the nucleus, a process essential for activating NF-κB target genes involved in inflammation and cell survival. PACs exerted a significant inhibitory effect on the MAPK signaling pathway, another critical conduit for transmitting extracellular signals to the nucleus, thus influencing cell proliferation, differentiation, and apoptosis [85,86]. PACs decrease the phosphorylation of key components of the MAPK pathway, namely p38, extracellular signal-regulated kinase (ERK), and c-Jun NH2-terminal kinase (JNK). By modulating these kinases, PCs can dampen MAPK signaling, reducing the expression of genes that drive inflammation and cell proliferation, two processes intimately linked to cancer progression [85,86]. These findings not only shed light on the molecular mechanisms underpinning the anticancer effects of PACs but also reinforce the potential of these compounds in the prevention and treatment of cancer. However, the specific molecular targets by which the isolated compounds exert their mechanisms of action remain to be studied. More research is needed on isolated proanthocyanidin compounds to identify the molecule responsible for their effects. This research gap highlights an opportunity to dive deeper into the bioactive components of proanthocyanidins, allowing a more precise understanding of their mechanisms of action and potentially uncovering targeted therapeutic applications. Other works have detailed the anticancer activity of other fruit peels. The review by Chukwuma et al. [5] emphasized the rich content of bioactive and therapeutic phytochemicals (flavonols and proanthocyanidins) in many fruit peels, including litchi, which exhibit notable anticancer potential. Naguib and A. Tantawy [87] evaluated the anticancer activity of various fruit peels, including pomegranate, orange, and lemon. Their findings demonstrated significant anticancer activity, particularly from pomegranate peel, which aligns with the properties observed in litchi peel. This comparison suggests that while litchi peel is effective, other fruit peels like pomegranate also offer anticancer properties, often related to their high antioxidant capacity.

### 5.6. Antihyperglycemic Activity

Contreras-Castro et al. [88] evaluated the antihyperglycemic properties of the peel from ‘Brewster’ litchis, harvested at two distinct stages of maturity, utilizing an oral glucose tolerance test conducted on Wistar rats. Peel from phase II litchis (mature fruits deemed suitable for export), tested at 25–200 mg/kg, lowered blood glucose levels in Wistar rats at 30 min during the oral glucose tolerance test. Litchi peel extract (10 mg/kg) combined with metformin (50 mg/kg), an antidiabetic drug, synergistically reduced blood glucose at 60 and 90 min in the oral glucose tolerance test with normoglycemic rats. Proanthocyanidins have been recognized for their potential antihyperglycemic properties, offering a promising approach to managing blood glucose levels. The mechanisms through which extracts containing proanthocyanidins exert their effects on blood sugar regulation involve intricate interactions with the insulin signaling pathway, investigations into these mechanisms have elucidated that extracts containing proanthocyanidins promote hypoglycemic effects primarily through the activation of the PI3K/Akt pathway, which plays a pivotal role in enhancing the translocation of the glucose transporter type 4 (GLUT4) to the cell membrane, thereby facilitating increased glucose transport into cells and enhancing cellular glucose uptake [70]. An additional significant aspect of proanthocyanidin action is its ability to reduce glycogenesis by inhibiting glycogen synthase kinase-3 beta (GSK-3β) [70]. However, it is important to note a significant limitation in the current research landscape: studies have primarily focused on proanthocyanidins, without delving into the effects of isolated compounds within the proanthocyanidin class. This gap points to the need for further investigation into individual proanthocyanidin molecules to fully understand their specific contributions and mechanisms of action of their antihyperglycemic activity. Besides litchi peel, other fruit peels have shown antihyperglycemic activity. Aziz et al. [89] showed that *Musa paradisiaca* leaf and fruit peel extracts improved impaired oral glucose tolerance and increased serum insulin levels in diabetic rats, indicating a potential similarity in function to litchi peel, even though each fruit peel exerts distinct phytochemical profiles and mechanisms of action. Additionally, *Citrus limetta* fruit peel normalized blood glucose levels and improved serum biochemical parameters in diabetic rats, suggesting another effective natural treatment for managing high blood sugar levels, comparable to the antihyperglycemic action observed with litchi peel [90].

## 6. Conclusions and Perspectives

This review underscores the critical importance of optimized processing techniques in maximizing the therapeutic potential of bioactive compounds from litchi peel. The comparative analysis of drying methods revealed significant impacts on the bioactive compound profiles, highlighting the necessity for methodical selection to preserve these valuable constituents. Similarly, the exploration of various extraction and purification strategies has illuminated the path toward enhancing the efficiency and specificity of isolating these compounds, a crucial step for their subsequent application in health and medicine. The biological activities identified through rigorous research corroborate the rich traditional lore surrounding litchi peel and underscore the fruit’s vast untapped potential in addressing contemporary health issues. It becomes evident that purification processes have successfully isolated specific compounds from litchi peel extracts, the bulk of pharmacological evaluations focused on the extracts rather than on these purified compounds. This gap in research highlights a significant opportunity for future investigations to delve into the pharmacological properties of the individual compounds isolated from litchi peel. The isolation of active compounds is crucial for characterizing the therapeutic potentials and mechanisms of action.

Litchi peel extracts and their active compounds have shown antioxidant effects with similar activity to that shown by reference drugs. The principal mechanism of action of the hepatoprotective, antiatherosclerotic, and cardioprotective effects shown by litchi peel extracts and their active compounds are related to their antioxidant activity. Anthocyanidins and flavonoids were the active compounds responsible for the pharmacological effects in litchi peel extracts.

The isolation and identification of bioactive compounds in litchi peel has unveiled a spectrum of beneficial activities for health, including antioxidant, antihyperglycemic, cardioprotective, hepatoprotective, antiatherosclerotic, and anticancer properties. Furthermore, the biological activities identified in litchi peel extracts suggest a broad spectrum of potential health benefits. Despite these promising findings, a significant limitation persists in the current body of research: most studies have focused on the collective effects of proanthocyanidins and other compounds that remain to be identified. This gap highlights an urgent need for further research on isolated compounds within the litchi peel to pinpoint the molecule(s) responsible for its various biological activities. Such targeted investigations could reveal the precise mechanisms of action and potentially unlock new therapeutic applications for these compounds.

Furthermore, the traditional use of litchi peel in Chinese medicine as a diuretic agent and for the treatment of diarrhea and parasitic infections suggests untapped potential for discovering additional biological activities [91,92,93]. Bridging the gap between traditional knowledge and modern scientific research could lead to identify novel compounds with significant health benefits.

The study of compounds isolated from the litchi peel presents a fertile ground for further research. One area ripe for exploration is the antidiarrheal activity of these compounds, a property recognized in traditional medicine but lacking extensive scientific validation. Investigating the specific compounds responsible for such activities can lead to the validation of traditional claims and potentially to the discovery of new pharmacological agents. Moreover, the pharmacological validation of activities reported in folk medicine extends beyond antidiarrheal properties. It encompasses a broader spectrum of therapeutic effects, including but not limited to anti-inflammatory, antimicrobial, and analgesic actions. Researchers can obtain insights into the mechanisms of action and the molecular targets by systematically identifying and isolating the bioactive compounds responsible for pharmacological effects. This can facilitate the development of novel compounds with enhanced efficacy and specificity for various pharmacological applications. Continued research into these compounds, guided by traditional medicinal knowledge and validated through modern scientific methods, holds the promise of new therapeutic agents for several health conditions. This endeavor will not only contribute to the understanding and appreciation of traditional medicine but also to the advancement of pharmacology and the development of novel therapeutic solutions.

Future studies should evaluate the effect of litchi peel consumption by humans in the development and progression of atherosclerosis, diabetes, and other cardiovascular diseases.

## Figures and Tables

**Table 1:** Method of drying, extraction, and purification of litchi peels.

Method	Drying Method	Solvent	Process	Purification	Result	Reference
Maceration	Lyophilized	Ethanol (68%)	1:20 (*w*/*v*) 59 °C 93 min	Using AB-8 macroporous resin and various chromatographic techniques	81.4% phenol content	[15]
	Lyophilized	Ethanol (70%)	50 °C 120 min	Column packed with AB-8 macroporous resin	Procyanidin content 95.85 ± 4.44%	[16]
	Lyophilized	Ethanol (70%)	50 °C 90 min		Procyanidin content 99.56 ± 1.2%	[17]
		Acetone	25 °C 240 min		The body weight of the mice was reduced by 12.3%	[9]
	Lyophilized	HCl 0.5 M	25 °C 12 horas	Amberlite XAD-7 resin column chromatography	Anthocyanin content 67.7%	[18]
		Acetone	1:10 (*w*/*v*) 25 °C 240 min		Strong antioxidant activity *in vitro*	[7]
	Oven-drying for 18 h at 60 °C	Methanol (70%)	1:10 (*w*/*v*) 40 °C 180 min		Total phenols content: 3.68 mg GAE/g-dw to 37.4 mg GAE/g-dw	[19]
	Air-drying for 2 h at 28 °C	HCl 0.5 M (in water)	25 °C 12 h		Total anthocyanin content of 18.6 mg/100 g	[20]
		Acetone (80%)	1:10 (*w*/*v*) 120 min 25 °C		Total phenolic content between 9.39 and 30.16 mg GAE/g FW	[21]
	Lyophilized	Saturated aqueous Na_2_CO_3_	pH 10 60 °C 6 h		Content of 75.06% total polyphenol, 13.98% total flavonoid, 5.51% total anthocyanin, and 28.53% total proanthocyanidin	[14]
	Liquid nitrogen	Cold aqueous ethanol (65%) with 0.5% sodium metabisulfite	1:10 (*w*/*v*) 30 min 4 °C		The DPPH radical scavenging activities of litchi phenolics were 73.09%.	[22]
	Hot air-drying (2–8 h), air-drying (5–7 days), steam blanching, and hot air-drying (2–5 h)28–80 °C	Ethanol (60%)	1:3 (*w*/*v*) 120 min 40 °C		Total phenolic compounds were reduced by 26.44% after air-drying for seven days. Hot air oven-drying at 40 °C reduced the total phenolic content by 12.03%, whereas drying above a temperature of 60 °C led to a reduction of phenolic groups by more than 40%.	[23]
		85% Ethanol:15% HCl	1:80 (*w*/*v*) 4 °C 120 min		The ethyl acetate fraction of flavonoids accounted for 83.1% of the total amount of flavonoids.	[24]
		Distilled water	1:20 (*w*/*v*) 30 °C 120 min	Gel filtration using a Sephadex G-50 column	Polysaccharide fractions showed antiradical activity (17–76%).	[25]
	Frozen in liquid nitrogen	Aqueous HCl solution 0.1 mol/L	1:10 (*w*/*v*) 25 °C 12 horas	Using Amberlite XAD-7 resin and Sephadex LH-20 columns	More than 1000 and 2000 mg of gallic acid equivalence per gram (mg GAE/g) were detected.	[11]
	Sun-drying	Methanol	1:10 (*w*/*v*) 4 days 25–32 °C	utilized a sequence of solvent fractionations, followed by silica gel column chromatography.	Antioxidant activity increased with increasing concentration for each sample (10–72%).	[26]
Ultrasound-assisted extraction	Oven-drying for 48 h at 60 °C	Methanol and aqueous acetone (70%, *v*/*v*)	1:5 (*w*/*v*) 20 min		Procyanidin content 2.2 (methanol) and 6.9% (acetone) (*w*/*w*)	[27]
	Oven-drying at 40 °C	Ethanol (70%)	1:10 (*w*/*v*) 15 min 45 °C		Total soluble phenols ranged from 51.3 to 102.1 g kg^−1^ DW	[28]
		Ethanol (20%)	200–400 w 60–100 min 10–20 mL/g		64.34 ± 2.65, showing strong antioxidant activity in baked goods.	[29]
	Oven-drying at 50 °C for 12 h.	Ethanol (40–90%) in 1% HCl (*w*/*w*)	1:20 (*w*/*v*). 30 °C 5–60 min 120–300 W		Ethanol concentration was the most important factor for cyanidin-3-rutinoside extraction.	[30]
	Oven-drying at 80 °C for 36 h.	Ethanol:HCl (85:15)	1:40 (*w*/*v*) 25 °C 30 min		The highest yields (30, 29, and 24%) of phenolic compounds were obtained when ultra-high pressure at 400 and 200 MPa and Ultrasound were applied.	[31]
Enzyme/Ultrasound-Assisted Extraction		Ethanol (20%)	1:15 (*w*/*v*) 50 °C 90 min		Process notably improved the yield of LPOPC, ranging from 8.25% to 14.20%, compared to the yield of less than 9% with only enzymatic treatment.	[29]
Ultra-high pressure assisted extraction		Aqueous ethanol	1:10 (*w*/*v*) 25 °C 10 min 100–500 MPa		ORAC (139.7 μmol TE/g) and CAA (34.21 μmol QE/g)	[32]
	Oven-drying at 80 °C for 36 h.	Ethanol:HCl (85:15)	1:40 (*w*/*v*) 25 °C 30 min 200–400 MPa		The highest yields (30, 29, and 24%) of phenolic compounds were obtained when Ultra-high pressure at 400 and 200 MPa and Ultrasound were applied.	[31]

**Table 2:** Compounds isolated from litchi peel, biological properties, and mechanisms of action.

Compound Name	Biological Properties	Mechanism of Action	Reference
**Polysaccharides**	Antioxidant activity	Chelation of metal ions	[63]
**Isolariciresinol**	Antioxidant activity	-	-
**Kaempferol**	Antioxidant activity	Reduce the production of free radicals and reactive oxygen species (ROS).	[64]
**Procyanidin B2**	Antioxidant activity	Interaction with reactive oxygen species (ROS). Activate the Nrf2 pathway. Inhibiting the MAPK/NF-κB pathway.	[65]
	Anti-atherosclerotic activity	Reduce levels of ROS.	[66]
**Epicatechin**	Cardioprotective activity	Stimulation of endothelial nitric oxide synthase (eNOS). Activation of signaling pathways such as the PI3K/Akt pathway. Inhibit stress-associated signaling pathways, including JNK/p38-MAPK Regulation of SUMO1-dependent modulation of SIRT1	[67]
	Hepatoprotective activity	Regulation of antioxidant enzymes. Direct scavenging of free radicals.	[68]
**Proanthocyanidins**	Antioxidant activity	Interaction with reactive oxygen species (ROS). Activate the Nrf2 pathway. Inhibiting the MAPK/NF-κB pathway.	[65]
	Anti-atherosclerotic activity	Expression of ATP-binding cassette subfamily A member 1 (ABCA1)	[69]
	Anticancer activity	NF-κB signaling pathway.	[66]
	Antihyperglycemic	Activation of the PI3K/Akt pathway. Inhibition of GSK-3β.	[70]
**Procyanidin A2**	Antioxidant activity	Interaction with reactive oxygen species (ROS). Activate the Nrf2 pathway. Inhibiting the MAPK/NF-κB pathway.	[65]
	Hepatoprotective activity	PI3K-Akt signaling pathway. HIF-1 signaling pathway.	[71]

## Data Availability

No new data were created or analyzed in this study. Data sharing is not applicable to this article.
